# Sustainable Probiotic Whey Protein Edible Films for Soft Cheese Quality and Shelf-Life Enhancement

**DOI:** 10.3390/foods15091570

**Published:** 2026-05-02

**Authors:** Charikleia Tsanasidou, Agathi Giannouli, Loulouda A. Bosnea, Antonia Terpou, Vasiliki G. Kontogianni

**Affiliations:** 1 Department of Agricultural Development, Agrifood and Natural Resources Management, School of Agricultural Development, Nutrition & Sustainability, National and Kapodistrian University of Athens, Evripos Campus, 34400 Chalcis, Greece; xaroulatsan@gmail.com (C.T.); aterpou@agro.uoa.gr (A.T.); 2 Dairy Research Department, Hellenic Agricultural Organization-DEMETER, Katsikas, 45221 Ioannina, Greece; agigiannouli@yahoo.gr (A.G.); bosnea@elgo.gr (L.A.B.); 3 Laboratory of Food Chemistry, Department of Chemistry, University of Ioannina, 45110 Ioannina, Greece

**Keywords:** WPC-edible films, probiotics, shelf life, simulated gastric conditions, sensory acceptance

## Abstract

Soft spread cheese is highly perishable, and conventional packaging offers limited protection against surface spoilage. Here, we present a sustainable, multifunctional solution: edible films made from whey protein concentrate (WPC), a valuable by-product of the cheese industry, incorporated with the probiotic *Lactobacillus acidophilus* LA5 (LA5). The objective of this study was to evaluate these films as active coatings for soft cheese, specifically assessing their physicochemical properties, probiotic viability during storage and simulated gastric transit, and their impact on cheese microbial stability and sensory quality over 60 days. Applied as active coatings on soft cheese stored at 4 °C for 60 days, these films were evaluated for their physicochemical properties, probiotic viability, microbial stability, and sensory acceptance. The incorporation of LA5 did not significantly alter film thickness (control: 0.20 ± 0.03 mm; test: 0.18 ± 0.02 mm), moisture content (control: 33.42 ± 0.54%; test: 32.34 ± 1.28%), or water solubility (control: 21.44 ± 1.14%; test: 22.89 ± 0.75%) (*p* > 0.05). However, mechanical properties were markedly modified: tensile strength decreased from 35.42 ± 5.38 MPa (control) to 6.04 ± 0.55 MPa (test), while elongation at break increased from 4.87 ± 0.93% to 68.23 ± 3.46% (*p* < 0.05), indicating a transition from rigidity to flexibility upon probiotic incorporation. The probiotic strain exhibited exceptional resilience, retaining 100% viability during simulated gastric exposure at both day 0 and day 30 of storage. During cheese storage, LA5 counts in test film-coated samples remained above the recommended therapeutic threshold (10^6^ cfu/g), starting at 7.44 ± 0.15 log(cfu/g) on day 0 and maintaining 6.56 ± 0.20 log(cfu/g) after 60 days. Critically, yeast and mold spoilage were delayed in probiotic-coated cheese, with detectable growth appearing only at day 60 (1.64 ± 1.34 log(cfu/g)), whereas uncoated cheese showed spoilage as early as day 28 (1.33 ± 1.62 log(cfu/g)). Sensory evaluation revealed no significant differences (*p* > 0.05) between the coated and uncoated samples for color, appearance, texture, flavor, or overall acceptability. By valorizing a dairy by-product into an active, probiotic-loaded edible film, this approach offers a sustainable, waste-reducing strategy that enhances cheese preservation while delivering added functional value—bridging the gap between food packaging and nutrition. Sensory evaluation (n = 8, preliminary) indicated no significant differences between coated and uncoated samples, but these results require confirmation with a larger, validated panel.

## 1. Introduction

Whey protein (WP) is a valuable by-product of the cheese industry with significant potential for valorization in food packaging as edible biopolymers. It is characterized by its high protein content (including β-lactoglobulin, α-lactalbumin, immunoglobulins, proteose-peptones, and lactoferrin), as well as its richness in minerals and vitamins. WP is distinguished by its lack of pathogens and toxic metabolites, its biocompatibility and biodegradability, and its generally recognized as safe (GRAS) status [1]. WP edible films can be consumed together with the product, functioning simultaneously as food and packaging, thereby enhancing the sustainability of the food system. Moreover, WP films combined with functional and health-promoting compounds may improve food shelf life while enhancing safety, nutritional value, and sensory quality [2].

Global demand for sustainable, functional packaging has catalyzed research into edible films and coatings that can both protect and enhance foods. Probiotics represent a promising example of bioactive compounds that can be incorporated into such films, enabling the delivery of high cell doses in a relatively compact matrix while providing protection against the harsh conditions of the upper gastrointestinal tract. The use of edible films as delivery systems for probiotics can effectively preserve probiotic viability, while these films can also serve as carriers of antimicrobial compounds, contributing to improved food safety and shelf-life extension [3].

Probiotics are live microorganisms that, when consumed in adequate amounts, confer health benefits to the host, regardless of the method of administration. To ensure these beneficial effects, it is generally recommended that the viable probiotic population, referred to as the “minimum therapeutic level”, remains at least 10^6^ cfu/g throughout the product’s shelf life [4]. However, probiotic viability is influenced by multiple factors (pH, acidity, oxygen, and water activity) during food processing and storage. A promising strategy to maintain the viability of probiotics during food processing and storage is their incorporation into edible films and coatings, which can provide a protective matrix during processing and throughout storage.

Cheese is considered an ideal medium as a probiotic carrier due to its high pH, composition (i.e., fat and protein), and solid structure. Soft cheese typically exhibits a pH range of 4.5–6.5, which is considered favorable for maintaining the viability of probiotic bacteria such as *Lactobacillus acidophilus* during refrigerated storage, compared to more acidic dairy products like yogurt (pH 3.8–4.2). Therefore, coating the final product with an edible film incorporated with probiotics may present an alternative approach for the development of food-grade matrix films that can simultaneously provide additional protection to cheese against physical damage and spoilage [5]. The incorporation of probiotic bacteria (*Lb. acidophilus* and *B. animalis*) into bacterial cellulose films and their application on white brined cheese (feta) have been investigated, demonstrating good probiotic viability (8.90 log(cfu/g)) [6]. The survival of probiotic bacteria has been shown to improve through encapsulation of the cells in pectin and sodium alginate matrices. The application of probiotic polysaccharide-based edible films on ultra-filtered soft cheese has also been proposed [7]. The results demonstrate that the probiotic bacteria *B. lactis*, *L. acidophilus*, and *L. casei* maintained viability after 45 days of storage (more than 8.00 log(cfu/g)), while the cheese coated with the edible films retained desirable chemical characteristics.

In a study by Guimaraes et al., 2020 [8], the antifungal properties of *Lentilactobacillus buchneri* incorporated into whey protein-based films/coatings were explored in semi-hard cheeses. Dopazo et al., 2022 [9], reported that films containing fermented whey with different lactic acid bacteria (LAB) strains inhibited the growth of three *Penicillium* species on cheese slices for up to 20 days under refrigerated storage conditions. In a similar study, cheeses wrapped with an edible film containing cheese whey fermented by a strain of *Levilactobacillus brevis* showed reduced fungal contamination during a 30-day ripening period [10].

The interactions between whey proteins and probiotic bacterial cells within edible films are governed by multiple physicochemical mechanisms. During film formation, bacterial cells may become embedded within the protein network through hydrogen bonding, electrostatic interactions, and hydrophobic forces between cell surface proteins and whey protein functional groups (e.g., –NH_2_, –COOH, and –OH). Li et al., 2025 [11], demonstrated that layer-by-layer coating of probiotics with whey protein isolate fibrils is primarily driven by electrostatic interactions and hydrogen bonding between hydroxyl or carboxyl groups of polysaccharides and amino groups in whey proteins. Similarly, Zhang et al., 2024 [12], showed that whey protein nanofibrils can be coated and anchored directly onto bacterial surfaces via biointerfacial supramolecular self-assembly, providing protection against environmental stress.

The incorporation of bacterial cells can act as physical fillers or structural disruptors, depending on cell concentration, size, and surface charge, potentially altering the mechanical properties of the resulting film. Specifically, bacterial cells may interrupt protein–protein cross-linking by competing for water molecules or by physically obstructing disulfide bond formation between whey protein monomers, leading to decreased tensile strength and increased elongation at break, as observed in previous studies with other probiotic strains. Sogut et al., 2022 [13], reported that incorporating *Lactobacillus acidophilus* into whey protein isolate films significantly decreased tensile strength and elongation at break values, demonstrating that probiotic cells act as structural modifiers within the protein matrix. Similarly, Karimi et al., 2020 [14], showed that *L. plantarum*-incorporated WPI films maintained good compatibility between the protein matrix and bacterial cells, with Fourier-transform infrared spectroscopy (FT-IR) analysis confirming no adverse chemical interactions. Understanding these interactions is critical for optimizing film formulations that balance mechanical integrity with probiotic functionality.

Soft spread cheeses are highly perishable due to elevated moisture, near-neutral pH (~4.5–6.5), and rich nutrient content that favors microbial spoilage, affecting many types of spoilage bacteria, yeasts, and molds. Conventional packaging offers passive protection (i.e., a non-interactive physical barrier that prevents direct contact with external contaminants but does not actively suppress spoilage organisms or add functional value) but lacks bioactivity. The incorporation of probiotics into WPC films may create active packaging systems capable of inhibiting surface spoilage, extending shelf life, and providing additional health benefits.

*Lactobacillus acidophilus* LA5 was selected for this study based on several criteria: (i) it is a well-characterized commercial probiotic strain with documented health benefits, including cholesterol reduction and anti-obesity effects [15]; (ii) it has demonstrated tolerance to acidic and bile salt conditions compared to other *Lactobacillus* species [2,3,7]; (iii) it has been successfully incorporated into various dairy matrices with high survival rates [5,8,10]; and (iv) its performance in WPC-based edible films has not been previously reported, representing a specific knowledge gap this study addresses. Specifically, Moura et al., 2016 [16], reported that LA5 maintained counts above 8.6 log cfu/g during refrigerated storage in dairy dessert while reducing LDL cholesterol in an animal model. Mumcu and Temiz, 2022 [17], demonstrated that LA5 exhibits higher acid and bile resistance compared to ATCC reference strains. Tratnik et al., 2008 [18], showed that WPC addition improved the growth and viability of LA5 in whey-based systems, supporting its compatibility with whey proteins. However, the application of LA5 in WPC edible films as active coatings for soft cheese has not been previously investigated.

Despite the growing body of research on probiotic edible films, several gaps remain. The specific interactions between WPC and probiotic bacterial cells during film formation and storage are not fully understood. Also, while various probiotic strains have been incorporated into edible films, comparative studies on strain-specific performance in soft spread cheese applications are limited. Finally, many studies have focused on short-term storage (≤30 days), with limited data on probiotic viability and film performance over extended periods (≥60 days).

We hypothesized that WPC-based edible films would serve as effective carriers for LA5, maintaining probiotic viability above the therapeutic threshold (≥10^6^ cfu/g) during 60 days of refrigerated storage while simultaneously delaying surface spoilage and preserving the sensory quality of soft cheese.

The objective of this study was to develop, characterize, and evaluate WPC-based edible films incorporated with LA5 as active coatings for soft spread cheeses. This study builds directly upon our previous work on herbal extract-activated whey protein films [19], which served as positive controls for antimicrobial activity. Here, we shift focus to a probiotic-based approach, evaluating feasibility and functionality rather than direct comparative efficacy. Specifically, this study aimed to: (i) characterize the physicochemical, mechanical, and structural properties of probiotic-loaded WPC films compared to control films using FT-IR, mechanical testing, and physicochemical analysis; (ii) assess the viability of LA5 during film formation, cold storage (60 days at 4 °C), and simulated gastric conditions; (iii) evaluate the effect of probiotic films on the microbiological stability (lactic acid bacteria, yeasts, and molds) of soft spread cheese throughout 60 days of storage; (iv) determine the sensory acceptability of cheese coated with probiotic films; and (v) investigate potential mechanisms of WPC–bacteria interactions influencing film properties.

## 2. Materials and Methods

### 2.1. Formulation of the Probiotic Carrier Films

Films were prepared according to Kontogianni et al., 2022 [19]. *Lactobacillus acidophilus* LA5 (hereafter referred to as LA5) was activated in MRS Broth and incubated at 37 °C for 72 h. The culture was then subcultured in fresh MRS broth under the same conditions (at 37 °C for 72 h). The resulting cell pellet was incorporated into the film-forming solution to achieve a final concentration of 10^7^ cfu/g (based on viable counts). Two separate inoculations of LA5 were prepared in the film-forming solution: one for the gastric simulation experiments conducted on films and another for the microbiological analyses of coated cheeses. These two inoculations were independent biological preparations. Regarding film characterization measurements, one inoculation of LA5 was prepared for the film-forming solution.

The following reagents were used: whey protein concentrate (WPC, 80% protein, Hellenic proteins SA, Ioannina, Greece), glycerol (≥99.5% purity, Sigma-Aldrich, Darmstadt, Germany), MRS broth and MRS agar (Oxoid, Basingstoke, UK), porcine pepsin (≥250 U/mg, Sigma-Aldrich, Darmstadt, Germany), and Ringer solution (Merck, Darmstadt, Germany). All chemicals were of analytical grade and used without further purification.

### 2.2. Enumeration of Probiotics in the Film Solutions

An aliquot of 1 mL from the film-forming solution was aseptically transferred into 9 mL of sterile Ringer solution to obtain the initial dilution [19]. Serial decimal dilutions were then prepared using the same diluent. Selected dilutions were plated on de Man, Rogosa, and Sharpe (MRS) agar and incubated at 37 °C for 72 h. After incubation, visible colonies were counted, and the results were expressed as logarithmic colony-forming units per gram of sample (log(cfu/g)).

### 2.3. Simulation of Gastric Passage of Films

The survival of the LA5 that was incorporated into the films was studied under simulated gastric conditions. The gastric simulation was performed at day 0 and day 30 (storage at 4 °C) according to Gagliarini et al., 2019 [20]. More specifically, the films were placed in plastic Petri dishes, hydrated in 5 mL PBS, and then exposed to 20 mL of simulated gastric juice by incubation at 37 °C for 3 h at 50 rpm under orbital shaking in a formal orbital shaker (Thermo Electron Corporation, Waltham, MA, USA).

Simulated gastric juice: NaCl 125 mM, KCl 7 mM, NaHCO_3_ 45 mM, and porcine pepsin 3 g/L. The pH was adjusted to 2.5 with a 0.1 M HCl solution. The gastric juice was freshly made for use on the same day.

Enumeration of viable lactic acid bacteria (LAB) of the film samples at 0, 1, and 3 h of gastric simulation time was carried out after centrifugation of the samples at 5000 g for 5 min as described by Gagliarini et al., 2019 [20]. LAB counts were enumerated on MRS Agar (37 °C for 72 h) and recorded as log(cfu/g). Moreover, the viability of the probiotic strain in films during gastric simulation was calculated according to the following equation:

Viability (%) = 100 × N/N_0_

where N is the initial number of LA5 (0 h of gastric simulation), and N_0_ represents the number of LA5 after 1 and 3 h of gastric simulation, respectively.

### 2.4. Determination of Physical and Mechanical Properties of Films

The thickness of films was measured using at least 6 points by a digital micrometer. The moisture content (MC) and water solubility (WC) of the film samples were determined according to procedures given in one of our previous studies [19]. Thus, we measured the tensile strength (TS), Young’s modulus (YM), and elongation at break (EB%) of the films.

### 2.5. Attenuated Total Reflection Fourier-Transform Infrared Spectroscopy (ATR-IR) Analysis

The interaction between the LA5 that was included in the films and film materials was analyzed using FT-IR. Infrared (IR) spectra of the films were obtained using a Jasco 4700 spectrometer (Jasco, Tokyo, Japan) equipped with a diamond attached to an ATR plate with a horizontal ATR accessory (Jasco ATR PRO ONE). The IR absorbance spectra were recorded in the range of 4000–650 cm^−1^ with a resolution of 4 cm^−1^, averaged over 64 scans, and then standardized using the air spectrum [21]. Measurements were performed in triplicate for each film formulation.

### 2.6. Edible Film Application on Soft Cheese

In sterile Petri dishes, thirty (30) g of soft cheese was weighted and classified into three groups (the first group consisted of only soft cheese without surface coating; the second group consisted of cheese coated with the control film without the probiotic strain; and the third group consisted of cheese coated with the enriched edible film, i.e., the test film, with the probiotic strain). We used the procedure previously described in [19]. All samples were stored at 4 °C for 60 days and collected for microbiological analysis at 0, 28, 42, and 60 days of storage.

### 2.7. Microbiological Analyses of Cheese

Ten (10) g of each cheese sample was homogenized with 90 mL of sterile Ringer solution (1/4 strength) to obtain the initial dilution. Serial decimal dilutions were then prepared, and 0.1 mL aliquots from the appropriate dilutions were spread onto the corresponding selective agar plates. Viable counts for lactic acid bacteria (LAB), yeasts, and molds were performed in duplicate at each sampling day. More specifically, LAB were plated on de Man, Rogosa, and Sharpe agar (MRS, 37 °C for 72 h), and yeasts and molds were plated on potato dextrose agar (PDA, 30 °C for 5 days). All counts were recorded as log(cfu/g).

### 2.8. Edible Film Application and Sensory Acceptance

The control and test films were applied to soft cheese to evaluate consumer acceptance. Soft cheese was weighted in sterile Petri dishes, classified into three groups as described above for edible film application, and was also used for the evaluation of sensory properties in terms of color, appearance, texture, flavor, and overall acceptability. The characterization of the sensorial quality of samples was performed at the beginning of the storage period. Eight panelists (ages 25–55; 5 females, 3 males) with experience in dairy product evaluation participated in the evaluation. While this sample size is common for exploratory sensory studies, it provides limited statistical power, and no formal panel validation (e.g., reproducibility or discrimination testing) was conducted. Therefore, the results should be considered preliminary. The panelists evaluated the organoleptic properties of the samples using a hedonic scale (1: dislike extremely, 9: like very much), according to Ceylan and Atosoy, 2022 [22].

### 2.9. Statistical Analysis

Conventional statistical methods were used to calculate the means and standard deviations of two simultaneous assays carried out with the different methods. Statistical analyses were performed by SPSS software 19.0 [23]. The mean differences between samples were compared using the one-way analysis of variance (ANOVA) and Tukey’s HSD (Honestly Significant Difference) test to determine statistically significant differences. Differences were considered significant at *p* < 0.05. The experiment was conducted with three independent biological replicates (n = 3), each representing a separate preparation of film-forming solution and probiotic culture. For each biological replicate, all measurements were performed in duplicate (technical replicates, n = 2). Thus, total replicates were n = 3 × 2 = 6 for each analysis, unless otherwise stated. Biological replicates account for variation between independent experimental runs, while technical replicates account for measurement variation within a single run. For datasets involving two factors (storage time and treatment), two-way analysis of variance (ANOVA) was performed using the General Linear Model in SPSS, including the interaction term (time × treatment). Where significant main effects or interactions were found, Tukey’s HSD post hoc test was applied. One-way ANOVA was used only for single-factor comparisons (e.g., film properties between the control and test films).

## 3. Results and Discussion

### 3.1. Effects of Edible Films on Probiotic Viability

The probiotic strain counts were 8.26 ± 0.56 log(cfu/g) in the film-forming solution and 7.44 ± 0.15 log(cfu/g) in the test films (day 0 of coating cheese). The reduction in the probiotic population might be attributed to the preparation of films containing probiotics. More specifically, the process comprises two deleterious steps for the probiotic bacteria: the osmotic effect of the film-forming solution and the dehydration effect of drying [24].

### 3.2. Probiotic Viability in a Simulated Gastric Condition

The ability of WPC films enriched with LA5 to carry probiotics through the gastrointestinal tract was evaluated by performing a simulation model. The films were hydrolyzed by pepsin in the simulated gastric juice. According to optical observations, the films disintegrated, and only small fragments of the films were still detectable after 3 h.

Table 1 presents the survival of LA5 in films during exposure to simulated gastric juice for 3 h at days 0 and 30 of storage (4 °C). Two-way ANOVA (storage time × gastric exposure time) revealed no significant main effects or interaction for LAB counts (*p* > 0.05 for all comparisons), confirming that LA5 viability remained stable under the tested conditions. The counts of probiotic strain were 6.82 ± 0.29 log(cfu/g) in the film-forming solution and 7.07 ± 0.14 log(cfu/g) in the test films (day 0 of gastric simulation). During storage at 4 °C, the probiotic population entrapped in the film slightly reduced from 7.07 (day 0) to 6.91 log(cfu/g) (day 30), but without a significant difference (*p* > 0.05). At day 0, LA5 viability remained at 7.07 log(cfu/g) after 3 h of exposure to acidic conditions. The viability rate of the probiotics after 3 h of simulation in gastric juice was 100.01%. At day 30, the cells of LA5 remained constant by 6.96 log(cfu/g) after a 3 h exposure to acidic conditions. The viability of LA5 showed no statistically significant reduction throughout the 3 h gastric simulation (*p* > 0.05). The calculated viability values (e.g., 100.01 ± 3.96% at 3 h, day 0) fall within the typical margin of error for plate counting (±0.5–1.0 log) and should not be interpreted as absolute 100% survival. Furthermore, the plate count method does not distinguish between fully viable and sublethally injured cells; thus, the reported values represent culturable cells rather than true counts. These values, which are within the margin of experimental error (±1 log cycle for plate counting), indicate that LA5 cells were not substantially inactivated under the tested conditions. However, it should be noted that the films disintegrated during gastric simulation, and the viability calculation reflects only culturable cells recovered from the digestion mixture; the possibility of cell injury or sublethal damage was not assessed.

The observation of near-100% viability during simulated gastric exposure should be interpreted with caution. While the results demonstrate that LA5 cells remained culturable after 3 h at pH 2.5 with pepsin, this finding may be influenced by methodological factors: (i) the films disintegrated during digestion, potentially releasing cells that were quickly enumerated; (ii) the simulated gastric model does not fully replicate the dynamic conditions of the human stomach (e.g., gradual pH changes, peristalsis, and food matrix effects); and (iii) the plate count method does not distinguish between fully viable cells and sublethally injured cells that may still form colonies on non-selective media. Therefore, while the WPC matrix appears to offer protective effects, the “100%” figure should be considered within the context of experimental variability (±0.5–1.0 log for plate counting).

The beneficial effects of probiotic bacteria are associated with their survival through the human gastrointestinal tract. The acidic condition of the stomach affects bacterial survival. Nevertheless, most bacteria are equipped with a physical or chemical barrier to resist acid exposure. No loss of LA5 viability was recorded due to the pH or to the action of gastric enzymes, proving that LA5 is resistant to the gastric conditions. The high recovery of viable cells in the film matrix after submission to gastric conditions could be due to the interaction of the probiotic strain with WPC, which would protect it from damage due to the oxidative and osmotic stress that occurs during drying and the film formulation process. This observation is in concordance with Soukoulis et al., 2014 [15]. Likewise, Gagliarini et al., 2019 [20], investigated whey protein–kefiran films as a carrier for *Lactobacillus paracasei* CIDCA 8339 and *Kluyveromyces marxianus* CIDCA 8154 and reported that these bacteria were able to recover after passing through the stomach and entering the neutral environment of the intestine.

The protective effect of the WPC matrix on LA5 viability during gastric exposure can be attributed to multiple mechanisms. First, whey proteins, particularly β-lactoglobulin, are known to bind bile salts and may also interact with pepsin, potentially reducing the accessibility of proteolytic enzymes to bacterial cell surfaces. Doherty et al., 2012 [25], demonstrated that β-lactoglobulin has a specific affinity for amphipathic compounds such as bile salts, and that whey protein matrices significantly enhance probiotic gastric tolerance compared to free cells. Similarly, Krunić and Rakin, 2022 [26], reported that protein-based encapsulation matrices contribute to both acid and bile tolerance of probiotics during simulated gastrointestinal conditions. Second, the protein matrix may create a localized pH buffering effect around bacterial cells due to the amphoteric nature of whey proteins (isoelectric point ~5.2), providing a microenvironment less acidic than the bulk gastric fluid (pH 2.5). This buffering capacity is supported by studies showing that whey proteins undergo conformational changes at different pH values, which can influence their protective functionality. Third, the physical entrapment of bacterial cells within the protein network may delay their exposure to gastric juice until the film begins to disintegrate, allowing time for the cells to mount acid stress responses (e.g., upregulation of H^+^-ATPase, production of acid-shock proteins). Gagliarini et al., 2019 [20], demonstrated that whey protein-based films effectively protect incorporated probiotics during gastrointestinal passage, with viable counts decreasing by less than 0.6 log cycle even after 57 days of film storage. The researchers confirmed that film disintegration occurs during gastrointestinal simulation.

### 3.3. Physical and Mechanical Properties of Films

The physical and mechanical properties of films are shown in Table 2. For comparisons involving only two groups (e.g., control vs. test film properties), one-way ANOVA was used. For multifactorial designs (treatment × time), two-way ANOVA was applied. The moisture content (MC) of the control films (33.42 ± 0.54%) and test films (32.34 ± 1.28%) did not differ significantly (*p >* 0.05). However, given the moderate statistical power of the study (see Section 2.9), the absence of a significant difference should be interpreted cautiously, and further studies with larger sample sizes are needed to confirm equivalence. Therefore, the inclusion of LA5 does not influence the MC of films. Our data are similar to those of Piermaria et al., 2015 [27]. They observed no significant differences in the moisture content of the kefiran films fortified with microorganisms. In contrast, Sánchez-González et al., 2014 [28], concluded that the presence of bacteria in the film matrix increased the moisture content of the film. Likewise, Sogut et al., 2022 [13], stated that the addition of probiotic bacteria significantly increased (*p <* 0.05) the moisture content of films made of whey protein isolate (WPI) and a mixture of WPI–carrageenan (75:25).

The water solubility (WS) of the films was not considerably modified by the fortification of the films with the probiotic strain. This result is in agreement with Kanmani et al., 2013 [29], who noted that the WS of the control and bacterial pullulan/starch blended films were not significantly different. Generally, the solubility of the film in water has a direct relationship with the moisture content, increasing with an increase in moisture content [29]. A film should show resistance to water in order to be utilized for the protection of intermediate- or high-moisture foods. All analyzed films remained intact after 24 h of storage in water, which is an indicator of a highly stable protein network.

The thickness values of all films were found below the required upper limit of 0.3 mm [30]. There were no differences detected in film thickness among the films. Similarly, Kalantarmahdavi et al., 2021 [30], observed no significant difference between the thickness of the control and the probiotic films. However, other researchers reported that films enriched with probiotic bacteria had higher thickness values [13].

Mechanical properties are related to the distribution and density of polymeric chains of the film matrix [30]. It is crucial for food coating materials to possess good mechanical properties. The mechanical properties of whey protein films are important to maintain their integrity during storage and handling. Young’s Modulus (YM), tensile strength (TS), and elongation at break (EB) are parameters that describe the film behavior under different conditions and expose changes in the film microstructure.

The incorporation of the probiotic strain significantly influenced (*p* < 0.05) the TS, EB, and YM of the films compared with the control (Table 2). Specifically, the presence of probiotic cells resulted in a marked decrease in TS and YM values, indicating a reduction in film rigidity and mechanical resistance, while EB increased substantially, suggesting enhanced film flexibility. Tensile strength corresponds to the maximum tensile stress that the film can sustain. The decrease in the TS value of the test films might be related to the interference of the probiotic strain in the structure of the protein network, interrupting the polymeric chain cohesiveness. Elongation at break is associated with the increase in film length from the initial point to the breakpoint, which is a key indicator of the film’s extensibility. The test films were characterized by a higher EB, indicating greater flexibility. Probably, the addition of LA5 increased the polymeric chain mobility due to weaker interactions of LA5 with the polymeric chain, leading to a more extensible and malleable film. Furthermore, LA5 reduced the intermolecular interactions of the polymeric chains and led to an increase in the lengths of the films due to stretching. ΥΜ represents the resistance of films to the elastic deformation. The higher YM values of the control films demonstrated that they had greater stiffness compared to the test films. A decrease in the TS and EB values of films incorporated with probiotics was noted in previous studies [27]. On the contrary, the mechanical parameters of films remained steady when microorganisms were included in the matrix in other studies [20].

The observed changes in mechanical properties—specifically the marked decrease in TS (from 35.42 ± 5.38 MPa to 6.04 ± 0.55 MPa) and the substantial increase in EB (from 4.87 ± 0.93% to 68.23 ± 3.46%) upon incorporation of LA5—can be explained by several interrelated mechanisms. Initially, bacterial cells may act as physical discontinuities within the protein network. During film formation, LA5 cells (approximately 0.5–1.0 μm in diameter) become embedded between whey protein polymers, disrupting the continuous protein–protein cross-linking network that typically provides mechanical strength. This “filler effect” has been previously described for bacterial cellulose and kefiran films containing probiotics [13,27].

Additionally, the bacterial cell surface is rich in exopolysaccharides, teichoic acids, and surface proteins that can interact with whey proteins through hydrogen bonding and electrostatic forces. These interactions may compete with native whey protein–whey protein interactions (e.g., disulfide bonds, hydrophobic interactions), effectively reducing the density of cross-links within the film matrix. The result is a more flexible, less rigid film, as evidenced by the increased elongation at break. Dianin et al., 2019 [31], demonstrated that incorporating *L. casei* into whey protein isolate films significantly altered mechanical properties, resulting in higher tensile strength (23.3 vs. 12.6 N) and lower flexibility (elongation at break: 5.27 vs. 45.4%), indicating that bacterial surface components interact with the protein matrix and modify cross-link density. Similarly, Sogut et al., 2022 [13], reported that probiotic incorporation significantly influenced the mechanical properties of whey protein-based films, with decreased tensile strength and elongation at break values, supporting the concept of bacterial cells as structural disruptors within the protein network. Vasiliauskaite et al., 2025 [32], further confirmed that LAB incorporation affects film properties through matrix interactions, noting that the impact of processing conditions on LAB survival provides insights into their adaptations and interactions within the film matrix.

Finally, the incorporation of bacterial cells may alter the water distribution within the film. Bacterial cells have high water-holding capacity due to their hydrated surface layers. This could plasticize the film matrix, increasing flexibility while reducing tensile strength, similar to the effect of glycerol as a plasticizer but through a different mechanism. Cazón et al., 2020 [33], demonstrated that water acts as a plasticizer in biopolymer films, with elongation values increasing due to the plasticizing effect of water molecules. Romano et al., 2014 [24], showed that incorporated components (fructo-oligosaccharides) had a plasticizing effect on film matrices, while also demonstrating via SEM that microorganisms can be incorporated without affecting film homogeneity. These findings support the hypothesis that bacterial cells, through their hydrated surface layers, may similarly alter water distribution and exert a plasticizing effect on the WPC film matrix.

### 3.4. Physical and Mechanical Properties of Films: Attenuated Total Reflection Fourier-Transform Infrared Spectroscopy (ATR-IR) Analysis

**Figure 1:**
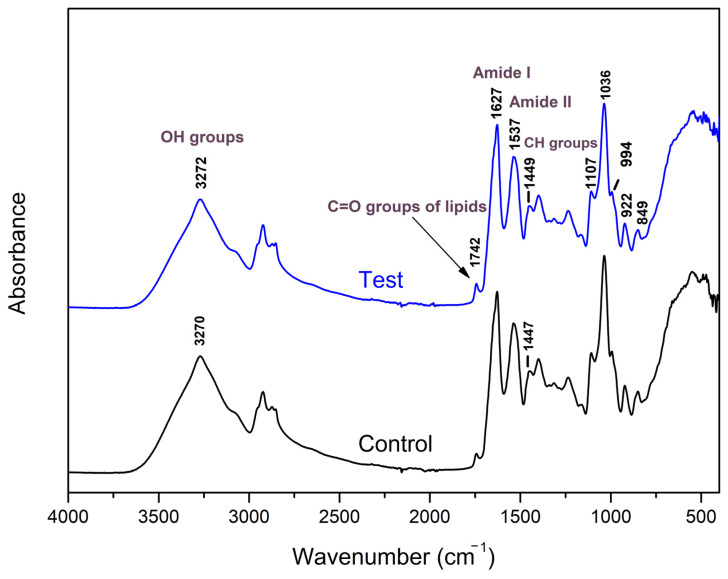
Attenuated total reflection Fourier-transform infrared spectroscopy (ATR-IR) spectrum of whey protein films incorporated with *L. acidophilus* LA5. Test = film with the probiotic strain; Control = film without the probiotic strain.

The ATR-IR spectra of the WPC-based films containing LA5 and the control film are shown in Figure 1. Both films showed identical IR absorption peaks in the fingerprint region of 4000–650 cm^−1^, indicating that incorporation of LA5 did not significantly alter the chemical structure of the films. In the same direction as the results of Kontogianni et al., 2021 [21], peaks in the spectral region at 800–1150 cm^−1^ are associated with the absorption bands of glycerol. Peaks appeared at 1030 cm^−1^ and 993 cm^−1^, which are associated with the amorphous phase and water-sensitive regions, respectively [34]. Strong absorption bands between 3000 and 3600 cm^−1^, corresponding to the stretching vibration of free and bound -OH and -NH groups, can also be observed. Bands at 1626 cm^−1^ and 1540 cm^−1^ are correlated with the amide I group and amide II group of proteins, respectively, and both are distinctive in whey proteins. Finally, the band at 1453 cm^−1^ corresponding to the stretching of -C-H in the CH_2_ groups and the signal at 1743 cm^−1^ that is associated with carbonyl stretching vibration can be recognized. The FT-IR analysis showed no major chemical structure changes (Figure 1), which suggests that the observed mechanical modifications are primarily physical (disruption of protein network continuity) rather than chemical (covalent modification of whey proteins) in nature. This is consistent with previous reports on probiotic-containing edible films [21,27].

### 3.5. Microbiological Changes in Coated Cheeses

The microbiological changes in all cheese samples were evaluated during 60 days of storage (Table 3). The viability of the incorporated strain was examined during the storage period of the coated cheese samples (60 days at 4 °C). As presented in Table 3, the initial probiotic concentration of samples coated with test films was between 7 and 8 log(cfu/g). Probiotic counts were maintained close to 7 log(cfu/g), with a slight decrease, until the 42nd day of storage. A loss of viability of ca. 1 log cycle, reaching 6 log(cfu/g), was recorded at the end of the storage period. The findings demonstrate that the probiotic cell counts were close to the values recommended to attain the beneficial health effects.

No spoilage or pathogenic bacteria were detected in any of the cheese samples until the 28th day of storage (Table 3). On the 28th day of storage, in cheeses without coating, 1.33 log(cfu/g) of yeasts and molds appeared and increased, without significant difference, to 1.88 log(cfu/g) on day 60. The yeast and mold count in cheeses covered with the control films was 1.62 log(cfu/g) on day 28 and remained constant throughout the end of the storage period. However, on day 42, the lack of yeast and mold detection may be attributed to the sensitivity of the method and the low yeast and mold count. In cheeses coated with test films, 1.64 log(cfu/g) of yeasts and molds appeared on the 60th day. However, visible mold growth was not observed in any of the cheese samples. Among the samples, at the end of the storage period, no significant changes were reported regarding the yeast and mold population. The spoilage of cheese is mainly due to the development of yeasts and molds. Two-way ANOVA (treatment × time) revealed no significant interaction effect on yeast/mold counts (*p* = 0.34), and the main effect of treatment was not significant at *α* = 0.05 (*p* = 0.07), which is likely due to the high variability and low statistical power. Nonetheless, the delayed onset of detectable spoilage (day 60 for test films vs. day 28 for uncoated cheese) suggests a biological effect warranting further investigation. Film enrichment with the probiotic strain retarded the growth of yeasts and molds and functioned as a possible delaying tendency in cheese spoilage. In the same direction, El-Sayed et al., 2021 [7], highlighted the antimicrobial effect of a probiotic film coated on soft cheese during 45 days of storage. According to the results, small invisible mold and yeast counts were detected on cheese at 45 days of storage. The antimicrobial property of the probiotic edible film was attributed to the antimicrobial activity of probiotics, which prevents pathogen growth and prolongs the shelf life of cheese.

### 3.6. Effect of Edible Film Application on Sensory Acceptance

**Figure 2:**
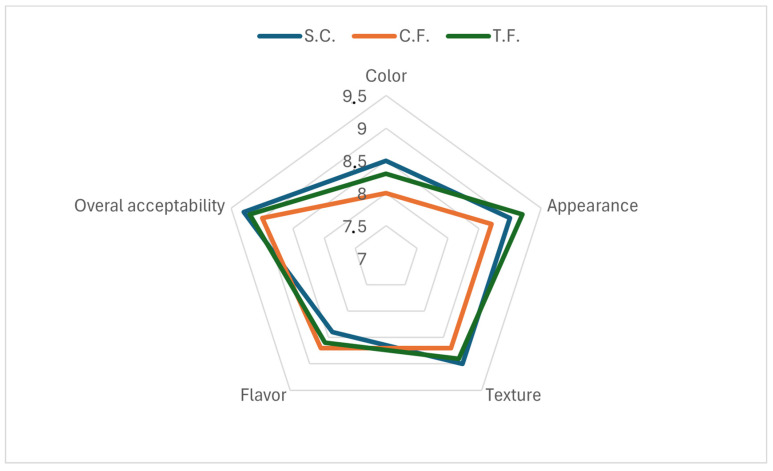
Radar chart displaying mean scores of the sensory properties of cheese samples. S.C. = soft cheese; C.F. = soft cheese coated with whey protein film; and T.F. = soft cheese coated with whey protein films incorporated with *L. acidophilus* LA5.

The sensory evaluation results of soft cheese samples in terms of color, appearance, texture, flavor, and overall acceptability are illustrated by the radar chart presented in Figure 2. Edible films should possess sensory characteristics that remain as neutral as possible, as they are often consumed along with the food products to which they are applied [35]. Therefore, the characterization of the sensory quality of the coated cheese samples is essential, as the incorporation of probiotics does not significantly affect the organoleptic properties of the cheese products [22]. The soft cheese scores for all the sensory properties were very close to the scores of cheeses applied with both films and the unwrapped soft cheese samples. There were no significant (*p* < 0.05) changes in any of the organoleptic properties evaluated. These scores indicated that all samples were acceptable from a sensory point of view. The panelists indicated that the addition of probiotics in the WPC films did not alter the odor of soft cheese, while they could not distinguish the cheese sample wrapped with the control film from the sample wrapped with the film with probiotics. Regarding the taste of the cheese samples consumed with the films, again, they could not identify any difference between the two film samples. They reported that the WPC films were tasteless and odorless, imbuing the cheese product with a faint sweet taste. In agreement with the present study, Ceylan and Atosoy, 2022 [22], reported that the addition of probiotics and prebiotics in sodium caseinate-based edible films did not significantly change any of the sensory properties evaluated. Their results indicated that cheeses coated with formulations containing probiotics had sensory property scores generally higher than the uncoated sample. In the same direction, El-Sayed et al., 2021 [7], verified that the coating of cheese enhanced its overall acceptability score. Given the small panel size (n = 8) and lack of formal validation, these sensory findings are preliminary. Larger studies (n ≥ 30) with trained, validated panels are needed to confirm the absence of sensory differences.

## 4. Conclusions

In this study, whey protein concentrate (WPC) edible films incorporating *L. acidophilus* LA5 were successfully developed and applied as active coatings on soft cheese stored at 4 °C for 60 days. The probiotic strain maintained viable counts above the recommended therapeutic threshold (≥10^6^ cfu/g) throughout the storage period, with counts of 6.56 ± 0.20 log(cfu/g) recorded at day 60. Additionally, LA5 incorporated into the films retained 100% viability during 3 h of simulated gastric exposure at both day 0 and day 30 of storage, suggesting that the WPC matrix may offer protection under acidic conditions. While the LA5 counts remained statistically unchanged during simulated gastric exposure, the interpretation of "100% viability" is limited by plate count variability and the absence of sublethal injury assessment. The incorporation of LA5 did not significantly affect film thickness, moisture content, or water solubility compared to the control films. However, mechanical properties were substantially altered, with decreased tensile strength and increased elongation at break, indicating that probiotic inclusion enhances film flexibility—a desirable attribute for conformable food coatings. Fourier-transform infrared spectroscopy confirmed that the chemical structure of the films remained unchanged upon probiotic incorporation.

Regarding microbial stability, the yeast and mold counts in probiotic-coated cheese samples remained below detectable levels until day 60, at which point they reached 1.64 ± 1.34 log(cfu/g). While these values did not differ significantly from those of the control-coated or uncoated samples at the end of storage (*p* > 0.05), the delayed onset of detectable spoilage (day 60 for test films versus day 28 for uncoated cheese) suggests a potential delaying effect that warrants further investigation.

Sensory evaluation revealed no statistically significant differences among any of the cheese samples across all attributes evaluated (preliminary, n = 8). However, due to the small panel size and absence of formal validation, these results should be interpreted with caution and confirmed in larger studies. This study has several limitations that should be addressed in future research. A positive control (e.g., a film containing a known antimicrobial agent such as rosemary or sage extract) was not included in the experimental design. This was a deliberate choice based on the progressive research strategy of our group. In our previous study [19], we developed and characterized whey protein films activated with rosemary and sage extracts as positive controls for soft cheese preservation, demonstrating significant antimicrobial activity and shelf-life extension. The present study builds upon that benchmark by exploring a different mechanistic approach—using probiotics as living bioactive agents—rather than comparing efficacy against plant-derived antimicrobials. Therefore, the absence of a positive control does not compromise the feasibility and functionality conclusions of this study. However, future head-to-head comparative studies between probiotic-loaded films and herbal extract-activated films are warranted to determine relative efficacy. The spatial distribution of LA5 cells within the WPC film matrix was not visualized; scanning electron microscopy (SEM) or confocal laser scanning microscopy would provide valuable insights into cell localization. Barrier properties (oxygen transmission rate, water vapor permeability) were not measured; these parameters are critical for predicting film performance for moisture-sensitive soft cheeses. The controlled release kinetics of probiotics from the film were not evaluated, and thermal stability (via differential scanning calorimetry or thermogravimetric analysis) was not assessed. Additionally, the study used a simplified in vitro gastric model and a laboratory-scale application format. While the kinetics of microbial viability were investigated, more sophisticated models incorporating multiple environmental factors (pH, water activity, and temperature fluctuations) would enhance predictive capability.

Taken together, these findings demonstrate that WPC-based edible films can serve as effective carriers for *L. acidophilus* LA5, maintaining probiotic viability during storage and under simulated gastric conditions while preserving the physicochemical and sensory qualities of soft cheese. Future research should include: (i) larger sensory panels (n ≥ 30) with formal training and validation; (ii) challenge studies with defined spoilage organisms; (iii) comprehensive film characterization (SEM, barrier properties, release kinetics, and thermal analysis); (iv) comparative studies including positive controls; and (v) validation under commercial storage conditions to confirm efficacy and scalability.

## Figures and Tables

**Table 1:** Viability of *L. acidophilus* LA5 in whey protein films during sequential exposure to simulated gastric juice (3 h) on the day of production and after 30 days of storage at 4 °C.

Time of Storage (Days)	Gastric Simulation (h)	LAB (log(cfu/g))	Viability (%)
0	0	7.07 ± 0.14 ^a^	100 ^a^
	1	7.08 ± 0.20 ^a^	100.19 ± 3.62 ^a^
	3	7.07 ± 0.20 ^a^	100.01 ± 3.96 ^a^
30	0	6.91 ± 0.08 ^b^	100 ^a^
	1	7.04 ± 0.07 ^ab^	101.93 ± 1.91 ^a^
	3	6.96 ± 0.03 ^b^	100.76 ± 1.26 ^a^

LAB: lactic acid bacteria. The results are the means ± standard deviation (n = 6). Different superscript letters within the same column indicate statistically significant differences (*p* < 0.05).

**Table 2:** The influence of the addition of *L. acidophilus* LA5 on the physical and mechanical properties of films.

Film	Thickness (mm)	MC (%)	S (%)	TS (MPa)	YM (MPa)	EB (%)
Control	0.20 ± 0.03 ^a^	33.42 ± 0.54 ^a^	21.44 ± 1.14 ^a^	35.42 ± 5.38 ^a^	1.93 ± 0.58 ^a^	4.87 ± 0.93 ^b^
Test	0.18 ± 0.02 ^a^	32.34 ± 1.28 ^a^	22.89 ± 0.75 ^a^	6.04 ± 0.55 ^b^	0.36 ± 0.04 ^b^	68.23 ± 3.46 ^a^

MC: moisture content; S: solubility; TS: tensile strength; YM: Young’s Modulus; and EB: elongation at break. The results are the means ± standard deviation (n = 6). Different superscript letters within the same column indicate significant differences (*p* < 0.05).

**Table 3:** Microbiological changes in cheese coated with whey protein films incorporated with *L. acidophilus* LA5 throughout the 60 days of storage at 4 °C.

	Soft Cheese log(cfu/g)		Soft Cheese + Control Film log(cfu/g)		Soft Cheese + Test Film log(cfu/g)	
Time of Storage (Days)	LAB	Yeasts/Molds	LAB	Yeasts/Molds	LAB	Yeasts/Molds
0	n.d.	n.d.	n.d.	n.d.	7.44 ± 0.15	n.d.
28	n.d.	1.33 ± 1.62	n.d.	1.62 ± 1.94	7.09 ± 0.10	n.d.
42	n.d.	0.65 ± 1.30	n.d.	n.d.	6.87 ± 0.52	n.d.
60	n.d.	1.88 ± 1.47	n.d.	1.61 ± 1.28	6.56 ± 0.20	1.64 ± 1.34

n.d. = not detected. LAB: lactic acid bacteria. The results are the means ± standard deviation (n = 6).

## Data Availability

The original contributions presented in the study are included in the article; further inquiries can be directed to the corresponding author.
